# Multimorbidity and AI-enabled health and social care: A methodological illustration of integrating large language models into qualitative analytic workflows

**DOI:** 10.1177/26335565261444423

**Published:** 2026-05-28

**Authors:** Callum Hill, Jacob Keast, Arun Dahil, Hajira Dambha-Miller

**Affiliations:** 1 Primary Care Research Centre, 7423 University of Southampton, Southampton, UK

**Keywords:** multimorbidity, qualitative analysis, artificial intelligence, large language models, social care

## Abstract

**Background:**

People living with multimorbidity often experience unmet social care needs, which can negatively affect wellbeing and increase pressure on health and social care systems. Artificial intelligence (AI)–enabled tools may support more timely and tailored responses to these needs. Large language models (LLMs) are emerging as tools to support qualitative research, although research detailing their integration into qualitative analytic workflows remains limited.

**Methods:**

We conducted a secondary thematic analysis of 75 qualitative interview transcripts involving people with multimorbidity and their carers. The dataset was coded according to an analytic framework of exploratory, interpretive, and integrative layers of meaning. The dataset was analysed according to two parallel analytic streams: human reflexive thematic analysis, and qualitative analysis using Claude Sonnet 4. Model outputs were iteratively reviewed and compared against manual thematic analysis for convergence and divergence.

**Results:**

Across the analytic workflow, twelve themes from the original human-led analysis were used as a reference framework for examining areas of alignment, extension, or divergence in LLM-generated interpretations. The LLM-assisted analysis highlighted shifts in analytic emphasis and candidate interpretive nuances, including emotive tone and latent cross-cutting concerns, while requiring human oversight to determine evidential grounding.

**Conclusions:**

We present a structured methodological illustration for integrating LLM-assisted outputs within qualitative analysis. Using convergence–divergence mapping, we examine how LLM-generated interpretations may function as an additional analytic lens that can support reflexivity, transparency, and analytic auditability in qualitative research applied within the context of multimorbidity.

## Background

Multimorbidity, defined as the co-occurrence of two or more chronic conditions, is a growing concern in UK healthcare.^1,2^ It is frequently associated with unmet social care needs, including support with housing, food, mobility, and everyday functioning, which are often poorly addressed due to fragmented systems, limited cross-sector integration, and pressures on the workforce.^3–5^ As a result, individuals with multimorbidity may experience poorer health outcomes, higher hospital use, and reduced quality of life.^6–8^ AI-enabled technologies, including decision-support systems and risk stratification models (distinct from generative language models such as LLMs), have been proposed as tools to support earlier and more personalised care for people with complex needs.^9–11^ However, their development and implementation must be informed by an understanding of stakeholder perceptions, particularly around trust, relevance, transparency, fairness, and potential bias.^12–15^ Research has highlighted the need to centre the voices of patients and carers in the co-design of such tools to ensure they are both acceptable and effective in practice.^16^

Alongside clinical applications, there is increasing interest in using AI tools to support the research process itself.^17^ Large language models (LLMs), such as Claude, are trained on extensive corpora of text and fine-tuned to perform a range of interpretive tasks, including summarisation, theme extraction, and conceptual clustering. Their potential to enhance qualitative research has gained attention, particularly in areas requiring the rapid synthesis of unstructured data.^18–20^ While early applications show that LLMs can aid in organising and synthesising qualitative data, the extent to which LLM-assisted outputs align with interpretive qualitative analysis, particularly around latent meaning, emotional tone, and cross-cutting patterns remains underexplored.^21–23^ This is especially relevant in health and social care settings, where subjective experience, relational dynamics, and power imbalances are central to meaning and interpretation.^24^

In thematic analysis, inductive approaches generate themes from the data, whereas deductive approaches apply pre-specified concepts or frameworks. Research on multimorbidity frequently relies on qualitative methods in order to explore topics such as lived experience, social care needs, and care fragmentation. As a result, methodological developments that support scalable qualitative analysis are of particular relevance to this field. There is limited empirical research detailing the integration of LLM-assisted analysis within qualitative workflows. To address this, we conducted a secondary analysis of interview transcripts involving people with multimorbidity and their carers, focusing on their views of AI-supported tools for social care.

The aim of this study was to explore how people living with multimorbidity and their carers perceive the use of AI-enabled tools to support social care needs, and to examine whether LLM-assisted analysis could complement human-led analysis. The primary contribution of this study is methodological. Using a qualitative dataset exploring multimorbidity and AI-supported social care, we provide a methodological illustration of how LLM-assisted outputs can be incorporated within a reflexive thematic analysis workflow, examining areas of convergence, divergence, and shifts in analytic framing between human and model-generated interpretations.

## Methods

### Study design

**Figure 1.**
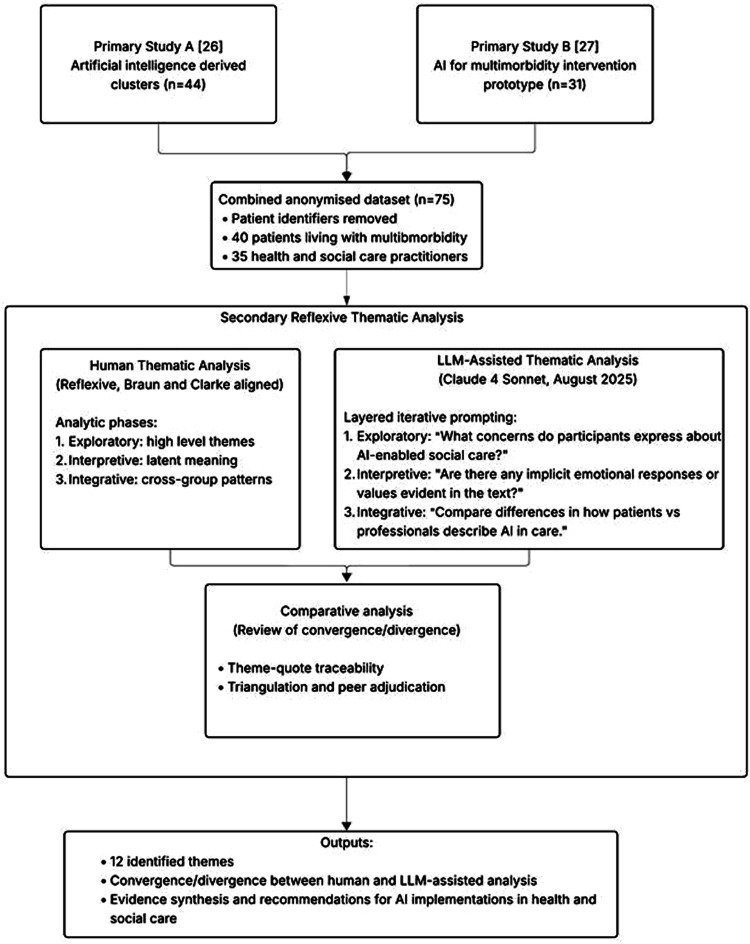
Primary data gathering and analytic workflow protocol.

We conducted a secondary thematic analysis of qualitative interview data collected as part of a broader project exploring the use of artificial intelligence (AI) to support people living with multimorbidity. This secondary analysis was designed as a methodological illustration of how human and LLM-assisted thematic analysis can be integrated in qualitative research applied to multimorbidity, using parallel analytic exploration across staged phases to examine areas of convergence, divergence, and interpretive risk. The secondary thematic analysis was reflexive, and Braun and Clarke aligned.^25^ The original studies employed a qualitative design, using semi-structured interviews to gather in-depth accounts from service users and carers, with the findings of these studies reported elsewhere.^26,27^ The analytic workflow used in this study is illustrated in Figure 1. This study adheres to SRQR (Standards for Reporting Qualitative Research) as reported in Appendix 1.

### Participants and recruitment

The primary analysis phase employed a combination of consecutive, purposive, and convenience sampling methods. Participants were recruited between September 2023 and March 2024 through voluntary sector organisations, social media platforms, academic networks, and local community groups across England. Eligible participants were adults aged 18 years or older who were living with two or more chronic physical or mental health conditions or caring for someone who was. All participants spoke English and gave consent for their data to be analysed according to the study protocol. Consistent with reflexive thematic analysis, recruitment continued until conceptual sufficiency was reached, defined as the dataset having the required depth and diversity to develop rich, well-evidenced themes which addressed our research questions.

A total of 75 individuals were interviewed: 40 people living with multimorbidity and 35 informal carers or professionals including general practitioners, social prescribers, community support workers, and wellbeing coaches. One participant agreed to interview but later did not participate. Characteristics of patients living with multimorbidity, and informal carers or professionals are shown in Tables 1 and 2 respectively.

**Table 1.** Participant characteristics.

Characteristic	N = 40^1^
**Sex**	
Female	29 (72.5%)
Male	10 (25.0%)
Prefer not to say	1 (2.5%)
**Age (years)**	47.5 (15.8)
**Ethnic origin**	
White – British	24 (60.0%)
Asian – Pakistani	4 (10.0%)
White – Other	3 (7.5%)
Asian – Indian	2 (5.0%)
Black – Caribbean	2 (5.0%)
Mixed background	2 (5.0%)
Other	2 (5.0%)
Black – African	1 (2.5%)
**Education**	
Other/unknown	12 (30.0%)
Postgraduate (Masters)	11 (27.5%)
A-level	5 (12.5%)
Undergraduate	5 (12.5%)
None/GCSE	4 (10.0%)
Postgraduate (PhD)	3 (7.5%)
**Number of long-term conditions**	4.3 (2.4)
**Interview length (minutes)**	48.6 (13.7)

^1^n (%); Mean (SD).

**Table 2.** Healthcare professional characteristics.

Characteristic	N = 35^1^
**Sex**	
Female	28 (80.0%)
Male	7 (20.0%)
**Age (years)**	47.3 (13.7)
**Ethnic origin**	
White – British	26 (74.3%)
Asian – Indian	4 (11.4%)
Black – African	1 (2.9%)
Black – Caribbean	1 (2.9%)
Mixed background	1 (2.9%)
Other	1 (2.9%)
White – Other	1 (2.9%)
**Role**	
Doctor/GP	9 (25.7%)
Manager/Leader	7 (20.0%)
Carer (paid/unpaid)	6 (17.1%)
Nurse/Clinician	6 (17.1%)
Other	5 (14.3%)
Social worker	2 (5.7%)
**Recruitment source**	
Friend/Colleague	13 (37.1%)
NHS/CRN/Local	8 (22.9%)
Other	7 (20.0%)
Work/Newsletter	5 (14.3%)
Online/Social	2 (5.7%)
**Interview length (minutes)**	41.1 (10.5)

^1^n (%); Mean (SD).

### Interviews

Interviews were conducted remotely via telephone or video call by trained qualitative researchers, as reported in the initial data collection studies.^26,27^ A single interview was conducted for each participant. Only the researcher and participants were present at interview. A semi-structured topic guide was used to explore participants’ experiences of managing daily challenges, their views on social care, and their reactions to a hypothetical AI-supported tool designed to assist with care planning. The guide included plain-language descriptions of AI and a vignette to prompt discussion. Interviews lasted between 21 and 102 minutes, were audio-recorded, transcribed verbatim, and fully anonymised. Field notes were kept in the primary analysis phase and documented alongside the transcript and analysis. All data collection and handling procedures were carried out in line with UK GDPR and ethical research standards.

### Qualitative analysis

Qualitative analysis was conducted by trained qualitative researchers in the primary data collection phase. Further details surrounding the initial data collection can be found in the respective primary analysis studies.^26,27^

In the secondary qualitative analysis phase, a qualitative researcher (HDM) reviewed the primary data collection according to the analytic framework. Analysis was conducted solely according to data from the transcripts. The analysis followed a guided inductive approach according to the study aims using staged prompts. Qualitative coding was conducted manually in the secondary analysis, without the use of coding software.

Prompts were initially developed to support summarisation and identification of potential codes. During early analysis, prompts were iteratively refined in response to preliminary outputs and emerging analytic priorities. Once the analytic approach was established, a consistent prompt structure was applied across transcripts. The secondary analysis was conducted as follows:

**1) Review of primary analysis coding:** We reviewed the inductive analysis conducted according to the two primary analysis studies. Inductive codes were reviewed iteratively according to three layers of meaning. At each layer, we generated and refined candidate themes and supporting extracts, progressing from descriptive patterns to more integrative synthesis. The following layers and corresponding prompts were utilised:
  (a) **Exploratory:** broad analysis and prompts to surface initial high-level themes.
  **Prompt:** “What concerns do participants express about AI-enabled social care?”
  (b) **Interpretive:** deeper analysis and prompts to elicit latent meanings, emotions, and value statements.
  **Prompt:** “Are there any implicit emotional responses or values evident in the text?”
  (c) **Integrative:** analysis and prompts to explore relationships and cross-cutting patterns across stakeholder types.
  **Prompt:** “Compare differences in how patients vs professionals describe AI in care.”

Further details of prompting structure can be found in Appendix 2.

**2) Parallel LLM-assisted analysis:** In parallel, we conducted an LLM-assisted analysis using Claude Sonnet 4. The same iterative prompts according to the three layers of meaning identified were used in the Claude analysis. Themes were derived from the output of the iterative analytic process.
**3) Comparative analysis:** We compared human and LLM-derived outputs at each analytic layer by mapping themes for conceptual equivalence (similar meaning with different labels), scope differences (broader or narrower framing), and emphasis shifts (foregrounding or backgrounding of issues). Convergence was recorded when the LLM recorded analogous or similar thematic framing to the human transcript analysis. Divergence was recorded when the LLM introduced interpretations not supported by the transcript evidence, conflated distinct concepts, or omitted central aspects present in the human analysis. Comparisons were performed by HDM and discussed with co-authors to resolve ambiguous mappings.

### Researcher characteristics and reflexivity

Researchers with expertise in primary care, health and social care implementations, and AI applications were involved in the development and analysis in this study. Several authors have experience in healthcare practice and AI applications in healthcare. We identified assumptions throughout the analysis in which participant data may conflict with researcher preconceived beliefs or ideas. These included the anticipated benefit of AI in healthcare implementations, and clinical experiential framing of the participants interviews. We actively sought negative cases for these emergent themes. Additionally, we utilised analyst triangulation for high-level interpretation of themes and areas of ambiguity. We recognise that LLM outputs may reflect biases present in their training data, highlighting the need for reflexive human oversight and triangulation when interpreting model-generated themes.

### LLM parallel analysis

Interview transcripts were provided to the LLM as anonymised plain text. To accommodate context limits while maintaining analytic coherence, transcripts were processed in segmented batches of approximately five interviews at a time. Segmentation was used solely as a practical processing step rather than as an analytic strategy, and themes were subsequently interpreted across the full dataset. Individual transcripts were not segmented.

For the parallel analysis, we used Claude Sonnet 4, a high-performing general-purpose LLM with long-context capabilities suited to transcript-based qualitative analysis. The analysis was conducted in August 2025. Model selection was informed by prior work demonstrating strong performance of Claude in a healthcare qualitative coding context, primarily using deductive approaches.^18^ The analysis was conducted on the Claude Web UI on a secure institutional workstation. Only fully anonymised transcripts were included in the analysis. No sensitive or identifiable information was provided to the LLM.

To minimise variability, the same model configuration and prompt structure were used throughout the analysis. Where outputs appeared ambiguous or unexpected, prompts were rerun for comparison. Final coding and thematic decisions were made by the research team based on the transcript data.

### Review of LLM outputs and non-retained interpretations

LLM outputs were reviewed independently by an experienced qualitative researcher (HDM) who compared them to themes from the original manual analysis. LLM outputs were documented alongside transcript segments and researcher notes. Outputs were treated as provisional analytic suggestions and were reviewed by the research team to assess their relevance and alignment with the transcript data. Analytic records were maintained to track prompt versions, model outputs, and subsequent coding decisions. Findings were retained for reporting if they aligned with human-derived themes, or offered candidate alternative framings that were verified as supported by the transcripts. All LLM outputs were verified to ensure all analysis and supporting evidence was grounded within the transcripts.

Where model outputs were judged to overstate, misattribute, or oversimplify meaning, these interpretations were not retained. For example, in response to the following participant statement:

*P02: “Yes, it can be, because there’s nothing I can do because that’s happened and you can only move forward, can’t you?”*

the LLM characterised the statement as reflecting a state of *“*quiet resignation*.”* Following review of the wider transcript context, this interpretation was judged to overstate the emotional tone and was therefore not retained.

Other examples of non-retained findings included cases of misattribution or oversimplification of meaning by the LLM. For example:

*P04: “when they go into hospital and come out, it all changes and then they get very confused about what they’re taking and what they’re not taking and when they’re having their next medication review.”*

was summarised by the model as “patients getting confused after discharge.”. During analytic review, this interpretation was considered an oversimplification of the participant’s account, and was not retained as thematic interpretation.

## Results

Seventy-five participants contributed to the study interviews: 40 people living with multimorbidity, and there were 35 health and social care professionals. Full participant characteristics are presented in Tables 1 and 2.

### Manual thematic analysis

Thematic findings are presented in three phases: exploratory, interpretive, and integrative. Each phase added progressive analytic depth and interpretive clarity. Quotes are attributed using anonymised participant IDs.

#### Exploratory: Surface-level themes and recurring concerns

The first layer of analysis identified eight overarching themes reflecting participants’ experiences of health and social care and their responses to the idea of AI-enabled tools.

**Uncertainty, risk, and AI scepticism**: There was interest in AI-supported tools but also doubt, particularly around privacy, transparency, and past negative experiences.

*“I don’t know what it’s doing with my info… is it helping or just profiling me?”* (P21)

**Power dynamics and communication gaps**: Many felt that their perspectives were not meaningfully heard by clinicians.

*“They just nod and write things down. I don’t think they’re really listening.”* (P07)

**Cognitive overload and fragmentation**: The effort of managing multiple conditions was described as overwhelming and isolating.

*“It’s the appointments, the meds, the forms… I lose track.”* (P33)

**Personalisation and future planning**: Participants wanted support tools that reflected their specific circumstances and could aid in long-term care planning.

*“Most tools assume I’m a stereotype. This one asked what I need.”* (P29)

**Barriers to digital access**: Digital exclusion was common and shaped by affordability, confidence, and cognitive challenges.

*“It’s all online now. I don’t even have a smartphone that works properly.”* (P12)

**Empathy and relational support**: Participants highlighted a need for emotionally responsive, human-centred care.

*“What I really need is someone who gets it. Not a printout.”* (P31)

#### Interpretive: Latent meaning, emotion, and value-laden responses

This second phase explored deeper emotional tone, embedded values, and how participants framed their experiences in moral and relational terms.

**Relational erosion and loss of trust**: Many accounts revealed a gradual breakdown in trust in professionals, often unspoken but implicit in tone and word choice. The experience of not being listened to was not just procedural, but was felt as a personal devaluation.

*“They’ve got their own script. I’m just filling time until they hit the next box.”* (P11)

**Emotional labour of managing multimorbidity**: Even where participants did not directly describe distress, their language often conveyed emotional exhaustion. Words like “juggle”, “keep track”, and “starting from scratch” appeared frequently, framing care as relentless effort.

*“During the pandemic, I lost my carer and couldn’t get help. I didn’t know who to call.”* (P18)

**Conditional hope**: Participants often expressed conditional willingness to try new tools, balancing hope with learned caution. This was particularly evident in repeated use of phrases like “if it actually helps” or “if it’s different this time.”

*“I’d give it a go… but I’ve tried lots of these things and they never seem to fit.”* (P08)

#### Integrative: Cross-cutting patterns across stakeholder types

The final phase examined patterns across participant groups, surfacing shared concerns and divergences between people with lived experience and those in professional or caring roles.

**Convergence on relational need**: Across groups, there was consistent emphasis on the value of human connection and relational continuity. Professionals as well as patients described frustration with transactional systems.

*“You can’t fix loneliness with a chatbot.”* (P40)

**Contrasts in optimism about AI**: Professionals and carers were generally more optimistic about AI as a means of coordination, while service users tended to frame their responses in emotional and experiential terms.

*“If it gives me more joined-up options, I’m all for it.”* (P46)

*“It sounds good, but I’ve been let down before. That doesn’t go away.”* (P25)

**Latent themes of exclusion and vulnerability**: While digital access was a visible barrier, the integrative analysis also surfaced less obvious exclusions, such as the assumption of a ‘standard user’, or the lack of emotional support in many systems. These concerns were voiced differently but echoed across interviews.

*“Everything’s designed for people who already know the system.”* (P16)

### Comparative analysis of manual and LLM-assisted findings

Using analogous phases and themes, we repeated the thematic analysis using Claude. Convergent and divergent findings are summarised in Table 3. The comparison here is intended to illustrate how LLMs may produce convergent or divergent findings when applied to qualitative research.

**Table 3.** Summary of convergent and divergent findings (by analytic phase).

Phase	Dimension	Convergent findings	Divergent findings
**Exploratory**	*Fragmentation/navigation*	Patients & HSCPs agree people often ‘project-manage’ care across fragmented services.	Alternative framing: reframed via the latent construct of cognitive overload.
	*Perceived empathy/support*	Reports of being dismissed; need for personalised, relational support.	Added nuance: foregrounded recognition/dignity in how dismissal is experienced.
	*Cognitive overload (explicit)*	Repetition, multiple appointments, and online admin recognised as burdensome.	Unified these as ‘cognitive overload’
	*Invisible labour (patients/carers)*	—	Made ‘invisible labour’ explicit (booking, translating between services, chasing referrals).
**Interpretive**	*Autonomy & dignity*	Desire to be seen as individuals and avoidance of labelling	Emphasised fears of depersonalisation with AI tools.
	*Attitudes towards AI*	Mixed optimism & scepticism; concerns about reliability and transparency.	Surfaced tension between hope and fear; ‘conditional hope’.
	*Power dynamics/asymmetries*	Often coded as ‘feeling unheard’.	Identified phrases such as ‘being passed around’ as hierarchy dynamics. Highlighted metaphorical language
	*Emotive language as data*	Coded as markers of fragmentation	Treated metaphors (e.g: ‘fighting fires’, ‘drowning’, ‘laying traps’) as affective analytic signals.
**Integrative**	*Coordination & relationships*	Participants prioritise joined-up care anchored in ongoing human relationships.	Carers/professionals stress structural barriers; patients stress emotional burden.
	*Exclusion & vulnerability*	Identified systemic procedural fatigue (e.g:‘no one listens’, ‘left to figure it out’).	—
	*Systemic metaphors*	Structural barriers documented.	Highlighted markers of disillusionment (e.g: ‘black box’, ‘maze’, ‘conveyor belt’).
	*Scepticism and perceptions of AI*	Interest in digital/AI tools tempered by privacy and lived experiences	Split into opacity vs deliverability; acceptance conditional on both being addressed.

#### Exploratory: Surface-level themes and recurring concerns

**Areas of convergence**

**Fragmentation in health and social care:** Both manual and LLM-assisted analysis converged on the fact that patients are sometimes required to “project-manage” their care. The theme of care fragmentation was identified in both patients and health and social care practitioners:

*“There's quite a lot of project management involved in sort of linking up my GP surgery with what my consultants are asking for”* (P18)

**Perceived lack of empathy:** Analysis utilising Claude highlighted the theme of perceived lack of empathy from professionals. Furthermore, the requirement for personalised, relational support was consistently identified as a prevailing theme within the LLM-assisted thematic analysis.

*“Chronic fatigue-type conditions or lots of other conditions... they just say it's anxiety and depression right and it can be very dismissive”* (P19)

**Areas of divergence**

**Reframing burdens as “cognitive overload”:** Manual coding identified the theme of repetition and fragmentation of care, frequently manifested in interviews through descriptions such as repeating histories to clinicians, managing appointments, and navigating online resources, LLM analysis converged on the idea of these experiences constituting the latent construct of cognitive overload. Although the theme of emotional burden and fragmentation was evidenced through manual coding, the LLM’s divergent contribution in this case was to unify and classify these ideas through the analytic frame of cognitive overload.

*“I would say that we get so many things popping up that we sometimes get a bit of click fatigue and just end up clicking through things without really properly taking them in.”* (P68)

**“Invisible labour” undertaken by patients and carers:** Manual analysis treated these as task lists; the LLM reframed them as invisible labour-essential coordination work that is uncounted and rarely acknowledged. It suggested that tasks such as managing appointments, translating between services, and following up referrals are not readily appreciated, but were made visible through carer and patient narratives.

*“I feel like I’m constantly chasing appointments. No one connects the dots.”* (P10)

#### Interpretive: Latent meaning, emotion, and value-laden responses

**Areas of convergence**

**Desire for dignity, autonomy, and recognition in care encounters:** Similarly to the manual thematic analysis, the LLM identified the recurring desire for dignity, autonomy, and recognition in care encounters. The LLM summarised patients’ views on autonomy, suggesting that “Many participants implicitly value being seen as whole people, not just cases or conditions.”. HSCPs expressed this theme with quotes such as:

*“As long as those people aren't defined by the groups that they've been put into because they're all individuals.”* (P44)

Exemplifying that HSCPs often share the desire to avoid “labelling” patients such that their identity is linked with their diagnosis.

**Practical and emotional ambivalence towards AI:** Both the model, and manual analysis suggested that there was widespread ambivalence towards AI. While some welcomed the potential for AI to support HSCPs in their role, others were more sceptical on its ability to replace human input.

*“The biggest problem is that the data that the NHS holds is very patchy… you could end up with conclusions that haven’t been tested out or aren’t right.”* (P67)

**Areas of divergence**

**Implicit power dynamics and asymmetries:** Analysis using Claude highlighted asymmetries between professionals and patients. It interpreted phrases such as “scripted conversations” and describing “being passed around” as markers of the perceived imbalance of power between patients and HSCPs. It highlighted language which could be identified as indicators as a loss of status or agency. This theme was not emphasised in the manual thematic analysis.

*“With the GP specifically… I end up going around in circles.”* (P38)

**Empathy as missing infrastructure:** Manual analysis suggested that participants desired more empathy in interactions with healthcare providers. LLM-assisted analysis reframed empathy as a prerequisite for interactions in social care. The model suggested that a significant proportion of participants viewed the relational/empathetic domain of social care interactions to be more important than any transactional value gained from AI implementations.

*“I could do with somebody who could understand or talk to. Not just a couple of pills.”* (P36)

### Integrative: Cross-cutting patterns across stakeholder types

**Areas of convergence**

**Coordination and relational continuity:** Both manual and LLM assisted analysis identified that patients, practitioners, and carers prioritise joined-up care anchored in ongoing, human relationships. The LLM output highlighted that carers and practitioners focused more on systematic barriers to implementing relational and coordinated care, whereas patients focused more on the emotional burden of care discontinuity and fragmentation.

*“A good GP would know their patient and what they struggle with.”* (P17)

**Exclusion, vulnerability, and procedural fatigue:** Manual and LLM analysis converged on the emotional and administrative toll of navigating care. Patients more often phrased this through a perspective of perceived vulnerability, with phrases such as “no one listens” and being “left to figure it out” frequently identified as markers of disillusionment. Procedural fatigue among HSCPs was more often expressed through administrative burden and capacity limitations.

*“People aren’t necessarily listened to about the support that they actually need.”* (P35)

**Areas of divergence**

**Systemic metaphors as signals of alienation:** The LLM analysis using Claude treated participants’ figurative language as markers of distrust of the care system. Metaphorical language with themes of opacity and mechanisation such as “black box”, “maze”, and “conveyor belt” were used in the LLM analysis to contrast participants’ views of the current system with the holistic care they desire.

*“You’re so busy trying to keep up with yourself that you don't have time to look after yourself, and then you end up laying bear traps”* (P47)

**Trust and Risk:** Manual thematic analysis identified ambivalence towards trusting AI tools, particularly surrounding data handling and AI. LLM-assisted analysis categorised ambivalence towards AI tools according to the categories of:

1) Opacity- which was highlighted in participant responses with regards to concerns surrounding profiling and data-usage.

*“I don’t know what it’s doing with my info… actually helping or just profiling me?”* (P21)

2) Deliverability- which was identified in responses in which participants questioned whether the AI interventions in social care would actually provide any benefits.

*“So many tools that promise and don’t deliver. I’d be cautious.”* (P08)

## Discussion

This study aimed to explore how people living with multimorbidity and their carers perceive the use of AI-enabled tools to support social care needs, while using the dataset to illustrate the integration of LLM-assisted outputs within reflexive thematic analysis by examining convergence, divergence, shifts in analytic emphasis, and interpretive risks.

Although many of the substantive themes identified (e.g., fragmentation, burden, relational continuity, and conditional trust in AI) are widely reported in existing multimorbidity literature, the primary contribution of this study is methodological. Rather than provide a formal evaluation of LLMs in thematic analysis, this study intends to demonstrate how model-generated interpretations can be used as an additional analytic lens.

Within this context, participants recognised both opportunities and limitations in AI-supported care. While many saw the potential for such tools to provide personalised guidance, support autonomy, and improve coordination across fragmented services, concerns remained regarding trust, accessibility, and the emotional limitations of automated systems. Findings by both manual and LLM analysis support existing literature that patients often feel that they must “project-manage” their own care, given the perceived fragmentation in care delivery.^28,29^

### Comparison to existing literature

Our analysis demonstrated that participants frequently expressed a desire for person-centred individualised care. This is supported by existing literature that suggests patients with long term conditions frequently feel dismissed or ignored by healthcare professionals.^30,31^ Power dynamics are a well-documented part of the health and social care system in the current literature, and if not managed appropriately can have negative consequences on patients’ experience.^32,33^ The emphasis on relational, personalised care throughout our analytic findings mirrors the consensus in multimorbidity research that care should be tailored to individuals, with meaningful patient inclusion.^34,35^

The use of LLMs in thematic analysis is an emerging area of research, in response to prior studies outlining the challenges of capturing the full scope and nuance of participant contributions in thematic analysis.^36–39^ As LLM capabilities develop, models such as Claude may offer a practical means of supporting qualitative analysis at scale by assisting with the organisation of data, surfacing candidate themes, and proposing integrative labels for further human interpretation.^40^ Thematic analysis is vulnerable to subjective influences, including researcher bias, and therefore benefits from reflexive approaches that make interpretation transparent.^41,42^ In this context, LLM-assisted outputs may function as an additional analytic lens highlighting patterns such as recurring concerns, emotional tone, or linguistic cues which can then be critically verified against transcript evidence.^23,43,44^

### Strengths and limitations

A key strength of this study is the use of a large, diverse qualitative dataset involving people with multimorbidity and informal carers. The inclusion of multiple lived experience perspectives enriched the analysis and enabled cross-cutting insights into shared and divergent needs and values. The transparent, staged prompting strategy applied to the large language model allowed for a structured and reproducible approach to LLM-assisted thematic analysis. By conducting exploratory, interpretive, and integrative layers of inquiry, the study demonstrates how LLM-assisted outputs can provide a complementary analytic lens, offering candidate alternative framings that can be assessed alongside reflexive human interpretation.

However, several limitations should be acknowledged. Claude is a general-purpose model and was not specifically trained on health or qualitative research data, which may limit its contextual sensitivity and cultural nuance. Furthermore, it should be noted that LLM outputs may reflect biases embedded into their training data. Although prompts and procedures were documented, LLMs are probabilistic models, meaning outputs may vary across runs and limit strict reproducibility. Sampling in the primary phases was conducted according to voluntary and network-based methods, which may introduce layers of bias to the demographics of participants recruited. The study was conducted within the context of the English health system, which may limit the transferability of findings to other contexts. Our study did not apply quantitative methods of agreement to compare performance in thematic analysis. Future work would incorporate a structured quantitative framework to measure convergence and divergence in the comparative analysis phase. Finally, while Claude offered candidate alternative framings, it should be noted that these could reasonably emerge through further human analysis of the data.

### Methodological implications and recommendations

Using this study as an example, we suggest several recommendations for implementation of LLMs within analytic workflows:

1) Treat LLM outputs as candidate interpretations: Consistent with emerging guidance on LLM-assisted qualitative analysis,^19,22^ our model outputs were most useful when treated as provisional analytic suggestions rather than definitive codes. LLM-generated summaries and thematic labels often provided alternative framings of participant narratives, prompting further reflection and verification by the research team.
2) Use LLMs to surface candidate alternative integrative findings: LLM-assisted analysis was particularly helpful in identifying potential relationships across large volumes of qualitative data. Model outputs frequently suggested integrative framings that linked concepts across interviews, supporting the identification of cross-cutting themes that could then be assessed through reflexive analysis.
3) Maintain reflexive human oversight: human reflexive judgement is central to qualitative analysis. While model outputs assisted in identifying themes, final thematic conclusions were derived from researcher determination.
4) Value of LLMs when exploring large datasets and scaling qualitative research: in an illustrative example of 75 transcripts, LLMs demonstrated the ability to rapidly process and synthesise participant data. We suggest that LLM-assisted analysis is of particular utility in large qualitative studies in which dataset size may present a barrier to analysis.

### Conclusion and future implications

This study demonstrates that people living with multimorbidity and their carers perceive AI-enabled tools as having the potential to support more personalised, coordinated, and proactive responses to social care needs. At the same time, participants expressed concerns about digital access, emotional disconnect, and the risk of impersonal care. Large language model-assisted additionally proposed alternative framings in thematic analysis of the transcript data. When used in partnership with human researchers, LLMs such as Claude can support more efficient and layered qualitative analysis without replacing critical, reflexive judgement.

This study therefore contributes a practical illustration of how LLMs can be integrated within reflexive thematic analysis using convergence-divergence mapping and human review of model outputs. Future research should continue to explore how LLMs can be used alongside human analysts to enhance rigour, speed, and depth in qualitative inquiry. Comparative studies evaluating outputs across different models with metrics of inter-rater reliability, co-analysis with public contributors, and the development of clear guidelines for LLM-assisted analysis in research frameworks for ethical and transparent deployment will be essential as these technologies become more prominent in the qualitative research landscape.

## Supplemental material

Supplemental material - Multimorbidity and AI-enabled health and social care: a methodological illustration of integrating large language models into qualitative analytic workflows Supplemental material for Multimorbidity and AI-enabled health and social care: a methodological illustration of integrating large language models into qualitative analytic workflows by Callum Hill, Jacob Keast, Arun Dahil and Hajira Dambha-Miller in Journal of Multimorbidity and Comorbidity.

Supplemental material - Multimorbidity and AI-enabled health and social care: a methodological illustration of integrating large language models into qualitative analytic workflows Supplemental material for Multimorbidity and AI-enabled health and social care: a methodological illustration of integrating large language models into qualitative analytic workflows by Callum Hill, Jacob Keast, Arun Dahil and Hajira Dambha-Miller in Journal of Multimorbidity and Comorbidity.

## Data Availability

Transcripts from this study are not publicly available due to the lack of participant consent for data sharing.*
