# Supplementary material for: Multimorbidity and AI-enabled health and social care: A methodological illustration of integrating large language models into qualitative analytic workflows
Source: J Multimorb Comorb. 2026 May 28;16:26335565261444423. doi: 10.1177/26335565261444423 (PMC13219934; doi:10.1177/26335565261444423)
Supplement: Supplemental material - Multimorbidity and AI-enabled health and social care: a methodological illustration of integrating large language models into qualitative analytic workflows [file sj-pdf-2-cob-10.1177_26335565261444423.pdf]

Appendix 2: Prompting Structure for LLM-Assisted Analysis

This appendix provides representative examples of the prompts used in the parallel large language model (LLM)-assisted analytic stream. All prompts were applied to fully anonymised interview transcripts. LLM outputs were treated as candidate analytic framings and were reviewed against transcript evidence during comparative analysis.

Phase 1 Prompt: Exploratory (Descriptive) Analysis

Aim: To surface initial, high-level themes and recurring concerns without imposing interpretive framing.

```
TASK: Exploratory thematic analysis.

Review the transcript below. Identify the main concerns, expectations, or
   experiences participants express regarding AI-enabled tools for
   supporting social care needs in the context of multimorbidity.


INPUT:
<TRANSCRIPT_TEXT>

OUTPUT:
- candidate descriptive themes
- supportive transcript evidencen for each theme
```

Phase 2 Prompt: Interpretive (Latent) Analysis

Aim: To explore latent meaning, emotional tone, and value-laden language.

```
TASK: Interpretive thematic analysis.

Re-examine the transcript with attention to:
- emotional tone (e.g., frustration, hope, scepticism),
- implied values (e.g., dignity, autonomy, fairness),
- underlying meanings that are suggested but not stated explicitly.

INPUT:
<TRANSCRIPT_TEXT>

OUTPUT:
- candidate interpretive themes.
- supportive transcript evidence for each theme
```

Phase 3 Prompt: Integrative (Cross-Cutting) Analysis

Aim: To identify cross-cutting patterns and differences across participant groups.

```
TASK: Integrative comparison across participant groups.

Compare how different groups (e.g. people living with multimorbidity,
   carers, health and social care professionals) describe AI-enabled
   tools in care.
```

```
INPUT:
<TRANSCRIPT_TEXT>

OUTPUT:
- candidate integrative themes.
- supportive transcript evidence for each theme
```
